# Deviants violating higher-order auditory regularities can become predictive and facilitate behaviour

**DOI:** 10.3758/s13414-023-02763-9

**Published:** 2023-08-02

**Authors:** Nina Coy, Alexandra Bendixen, Sabine Grimm, Urte Roeber, Erich Schröger

**Affiliations:** 1 Wilhelm-Wundt-Institute of Psychology, University of Leipzig, Leipzig, Germany; 2 Max Planck School of Cognition, Leipzig, Germany; 3 Cognitive Systems Lab, Institute of Physics, Chemnitz University of Technology, Chemnitz, Germany; 4 Physics of Cognition Lab, Institute of Physics, Chemnitz University of Technology, Chemnitz, Germany

**Keywords:** Predictive processing, Deviant repetition, Oddball, Auditory perception, Higher-order regularity

## Abstract

The human auditory system is believed to represent regularities inherent in auditory information in internal models. Sounds not matching the standard regularity (deviants) elicit prediction error, alerting the system to information not explainable within currently active models. Here, we examine the widely neglected characteristic of deviants bearing predictive information themselves. In a modified version of the oddball paradigm, using higher-order regularities, we set up different expectations regarding the sound following a deviant. Higher-order regularities were defined by the relation of pitch within tone pairs (rather than absolute pitch of individual tones). In a deviant detection task participants listened to oddball sequences including two deviant types following diametrically opposed rules: one occurred mostly in succession (high repetition probability) and the other mostly in isolation (low repetition probability). Participants in Experiment 1 were not informed (naïve), whereas in Experiment 2 they were made aware of the repetition rules. Response times significantly decreased from first to second deviant when repetition probability was high—albeit more in the presence of explicit rule knowledge. There was no evidence of a facilitation effect when repetition probability was low. Significantly more false alarms occurred in response to standards following high compared with low repetition probability deviants, but only in participants aware of the repetition rules. These findings provide evidence that not only deviants violating lower- but also higher-order regularities can inform predictions about auditory events. More generally, they confirm the utility of this new paradigm to gather further insights into the predictive properties of the human brain.

## Introduction

Prior information and previous experience are believed to shape cognitive processing by allowing the human brain to infer future states of the world (Friston, 2005; Knill & Pouget, 2004). In the human auditory system, they supposedly inform the building and maintenance of internal representations of the auditory environment which are constantly validated by comparing predictions inferred from them with the actually received sensory input—the difference between the latter two is termed prediction error (Garrido et al., 2009; Winkler, 2007). While it is not yet fully understood what exact form these internal models take and how failed predictions affect their content (Winkler & Czigler, 2012), it is well documented that different forms of auditory regularities are extracted by the auditory system and that deviations from them result in prediction error (Paavilainen, 2013; Winkler, 2007). Auditory regularities pertain to the recurrence of a specific sound or physical feature value (lower-order regularities; e.g., all sounds have the same pitch) as well as to some common rule that governs stimuli and their relationship (higher-order regularities; e.g., sounds have varying pitch but are arranged in pairs with the same direction of within-pair pitch change). Representing auditory regularities serves the formation of perceptual objects in auditory scene analysis (Bendixen, 2014; Winkler, 2007; Winkler & Schröger, 2015) and more generally, reduces processing requirements for incoming stimulation: The better the internal models map the auditory environment, the more accurate predictions about the imminent future become, which boosts processing efficiency. Evidence for these processing principles largely comes from variations of the oddball paradigm, in which a frequent standard stimulus occasionally is replaced by a rare deviant stimulus, which violates an established standard regularity (e.g., by having a different pitch from the standard, or by violating a standard intersound relationship). While the deviant is a potent tool to probe the extraction of auditory regularities of varying complexity, it is surprisingly underspecified when it comes to what value it contributes to the internal model itself. The deviant provides precise information about the next stimulus—in the classic oddball paradigm, a deviant typically occurs in isolation and is, thus, always followed by a standard stimulus. This poses the question whether this information that is carried by the deviant is incorporated in the internal models and used to infer local predictions—namely, in the classic oddball paradigm that when encountering a deviant, the next stimulus will be a standard. Although there could be a local prediction (“a deviant is always followed by a standard”), in the classic oddball framework there is no easy dissociation from other effects. Namely, there could be a global prediction resulting in the same expectation as the local prediction (“the most probable stimulus in any trial is a standard”). Furthermore, while there are several studies that have shown differential processing (Ahveninen et al., 2000; Berti, 2008; Parmentier & Andrés, 2010; Roeber et al., 2003) of the standard following a deviant compared with the “regular” standards (i.e., standards following standards), the comparison between a standard following a deviant and a standard following a standard might not be ideal. This is because there is a different amount of stimulus-specific adaptation in standard-standard-standard compared with standard-deviant-standard micro-sequences (Ulanovsky et al., 2004). Alternatively, the deviant might have transiently weakened the global prediction of the standard stimulus. To circumvent these issues, several studies (Berti, 2008; Parmentier et al., 2011; Rosburg et al., 2018; Todd & Mullens, 2011; Todd & Robinson, 2010) introduced deviant repetition to the stimulation: Instead of isolated single deviants within the standard stimulation, a deviant is followed with a certain probability by a second deviant. Indeed, effects on behaviour were reported that cannot be explained by a global regularity-based prediction alone. In an auditory oddball study, deviants were always repeated, and behavioural distraction (longer response times relative to regular standards) substantially decreased from first to second deviant (Berti, 2008). In a crossmodal oddball study (Parmentier et al., 2011) in which deviants had a high conditional probability to repeat, behavioural distraction was observed for first but not for second deviants. On the rare occasion that a standard directly followed a single deviant, the distraction effect was of similar magnitude as the response to a first deviant. However, this manipulation approach does not entirely exclude alternative explanations for the observed effects: The successive deviant presentation could yield the deviant detection system refractory, or a first deviant might reduce confidence in the global regularity of standard repetition. To address this, Sussman and Winkler (2001) manipulated deviant repetition probability between different experimental blocks in an electrophysiological study, and observed a stronger reduction of prediction error processing for the second compared with the first deviant when deviant repetition was more likely. This excludes the refractoriness-based explanation, but it does not provide a solution to the issue of global confidence in standard repetition, since the manipulation was applied across blocks, allowing for different global regularities to be formed.

Recently, we proposed a further variation of the classic oddball paradigm which contrasts different repetition probabilities within an ongoing stimulation (Coy et al., 2022): Two diametrically opposed deviant repetition rules were consistently associated with two deviants of differing pitch throughout the experiment—one deviant was more likely followed by a second deviant than a standard (high repetition probability) and the other deviant was more likely followed by a standard than by a deviant (low repetition probability). Indeed, although participants were not informed about the existence of the two deviant repetition rules, the repetition rules differentially affected performance in a simple deviant-detection task: Response times decreased from first to second deviant when deviant repetition probability was high but not when it was low. While there was no effect of repetition rule on the hit rates (possibly because they were near ceiling), false-alarm rates increased in response to standards following single deviant presentations for high compared with low deviant repetition probability. These findings provide further evidence that deviants are integrated into the internal predictive models, facilitating precise expectations about auditory events in the imminent future. To probe this further, here, we transfer the paradigm by Coy et al. (2022) to a higher-order auditory regularity.

For lower-order regularities, a direct association can be made of a particular stimulus, or a specific low-level feature, with a specific response—for instance, pressing a button when encountering either a high- or a low-pitched sound (deviant) but not in response to the medium-pitched sound (standard). For higher-order regularities, a response strategy that links a particular sound with a specific response is not as useful, as first-order features (e.g., pitch) are constantly changing. Thus, the pitch of any single sound is uninformative as to the appropriate response. In these instances, it is the relation between features of (at least) two successive sounds that needs to be mapped to a corresponding response—for instance, pressing a button if the pitch of two sounds is different, and not responding with a button press if they have the same pitch. There is evidence that deviations from a higher-order regularity are not only processed as prediction error, as indexed by electrophysiological indicators (Paavilainen et al., 2003; Saarinen et al., 1992; van Zuijen et al., 2006), but that they can be detected and influence behaviour even when task-irrelevant (Schröger et al., 2007). Therefore, the question poses itself whether extraction and application of predictions based on deviants generalize from regularities based on first-order stimulus features to higher-order regularity contexts. If the ability to encode deviant repetition rules was limited to contexts investigated in preceding studies (Coy et al., 2022; Sussman & Winkler, 2001) in which a specific response can be linked to a specific low-level feature value (e.g., pitch), then this would cast doubts on formulating this ability as a general principle of internal auditory models. In contrast, it would be a strong argument for generalizing deviant-based predictions as an overarching processing principle, if repetition rules can be encoded and inform behaviour when they are embedded in a higher-order regularity (here, the direction of within-pair pitch change) while the absolute feature values constantly change and are thus uninformative of the appropriate response.

To examine this generalizability, we implemented the aforementioned variation of the oddball paradigm within a higher-order regularity stimulation protocol (Saarinen et al., 1992): All stimuli consisted of two short tones randomly varying in pitch between trials. For standard sounds, both tones of a given pair were of the same pitch (constant pair), while deviant sounds consisted of two tones differing in pitch. Specifically, one deviant type had an “ascending” structure (second tone higher than the first) and the other deviant type had a “descending” structure (second tone lower than the first). In correspondence with our oddball paradigm variation (Coy et al., 2022), each deviant type (ascending/descending) was associated with one deviant repetition rule (deviant repetition probability high vs low). As it is well known that processing is facilitated when targets are predictable (Los & Schut, 2008), observed performance differences in the target detection task would be taken as evidence that the underlying predictive information was extracted. Hypotheses were based on the notion that deviants are incorporated into the predictive models and translate into behaviour: When a prediction activated upon encountering a first deviant is matched by the incoming sensory input (prediction confirmation), processing should be facilitated in the form of either or both increased accuracy and reduced response times. When a prediction is mismatched by the incoming sensory input (prediction fails), processing should be impeded in the form of either or both reduced accuracy and prolongation of response times. To elaborate, we expected that when a deviant is followed by a second deviant, hit rates should be higher when repetition probability is high (predictable deviant repetition) compared with low (unpredictable deviant repetition; Hypothesis 1). Further, when a first deviant is followed by a standard sound, we hypothesized that false-alarm rates should be lower for low-repetition probability (predictable nonrepetition) than for high-repetition probability (unpredictable nonrepetition; Hypothesis 2). Lastly, we expected a response time facilitation in deviant repetition from first to second deviant when repetition probability is high but not when it is low (Hypothesis 3). As deviant-detection performance might be generally modest for higher-order regularities (Schröger et al., 2007), effect sizes might be smaller compared with the first-order regularities employed in the preceding study (Coy et al., 2022).

## Experiment 1

### Materials and methods

#### Participants

Participants (mean age *M* = 22.3 years, ranging from 18 to 38 years) were recruited online, and were compensated for their participation either in the form of payment (8€/h) or course credit. Protocol and procedures were in accordance with the Declaration of Helsinki and approved by the Ethics Advisory Board at Leipzig University (RF: 2021.04.08_eb_83). Forty-eight people participated in Experiment 1, which was implemented in an online (i.e., web-based) setting. The sample size was determined so that for a paired one-tailed *t* test there was approximately 80% power to detect an effect of at least medium size (Cohen’s *d*_z_ ≥ .5) given an alpha of 0.5%. Note, that the alpha level was lowered to 0.5% to reduce the probability of false positive findings (Benjamin et al., 2018). One additionally tested participant was excluded because they failed to detect at least 50% of either one of the two target types in Position 1 (first deviants) and responded to more than 10% of the regular standards with a button press (i.e., a false alarm). All included participants reported normal or corrected-to-normal vision and hearing.

#### Procedure and data acquisition

The experiment was programmed in the JavaScript library jsPsych (Version 6.3.0; de Leeuw, 2015) and run on a JATOS server (Lange et al., 2015) hosted at Leipzig University. Participants received a copy of the information sheet for the experiment and a personal single worker link by e-mail to start the experiment from their own home computer, using either a Mozilla Codebase or Chromium-based browser. They were asked to wear cable-bound headphones with jack-plug (no Bluetooth, no usage of noise-cancelling function, ideally no USB) and to adjust the sound intensity themselves by listening to a short test sequence, so they could hear the sounds well, yet at a comfortable level.

We asked participants to listen to sound sequences consisting of tone pairs of varying pitch while gazing at a black fixation cross against a white background. Tone pairs could either be composed of two identical tones (constant pair) or two tones differing in pitch (ascending or descending pair). We instructed participants to respond as quickly as possible with a key press (space bar) whenever they detected a tone pair consisting of two different tones (i.e., ascending or descending tone pair). Feedback of task performance (hit rate, false-alarm rate, and average response time) was given at the end of each block. There was a short training block (55 trials), on which participants received direct feedback on the target detection (fixation cross temporarily turned green for hits, red for false alarms and into a red minus for misses). The training block could be repeated as often as participants wanted before proceeding to the main part (42 of the participants performed one, five performed two; one performed three training blocks).

As deviant repetition rules might only be extracted and applied successfully if participants are able to recognize a given deviant type in the first place, there was a control task after the main part to probe whether participants are in principle able to discriminate ascending from descending tone pairs. In two blocks of 40 trials each, ascending and descending tone pairs (50% each; 1,200-ms trial duration) were presented in randomized order. Participants were instructed to press the space bar whenever they detected an ascending tone pair. The control task was followed by a brief survey, inquiring about potential strategies participants had used during the experiment, whether they had noticed any rules that sounds had followed, and general feedback. The whole experiment, including instructions, training, control task, debriefing and some breaks, took approximately one and a half hours.

#### Stimuli and design

On each trial, a tone pair was presented consisting of two 50-ms sinusoids (5-ms rise/fall time) separated by a 100-ms silent interval. Absolute pitch of the first tone in each pair was randomly sampled on each trial from a uniform pool between 400 and 900 Hz (in steps of 10 Hz). Relative to the first tone, the pitch of the second tone increased by 25% for ascending tone pairs and decreased by 25% for descending tone pairs. For constant tone pairs the pitch of the second tone was identical to the first. All tone pairs (200-ms total duration) were created in MATLAB and exported as *wav* files. These sound files were downloaded to local memory on each participant’s computer at the start of the experiment (*jspsych-preload* option). Stimuli were presented with the *jspsych-audio-keyboard-response* plugin, which was modified to register all responses within a trial and not only the first key press. In the main part, stimulus-onset asynchrony (SOA) was jittered by randomly sampling on each trial from a uniform pool between 950 and 1,150 ms (in steps of 20 ms). The jittered SOA ensured that a first deviant was foremost informative as to the identity of the next sound (“what” prediction), rather than the time point of its occurrence (“when” prediction). In the control task after the main part, SOA was uniformly 1,200 ms.

**Fig. 1:**
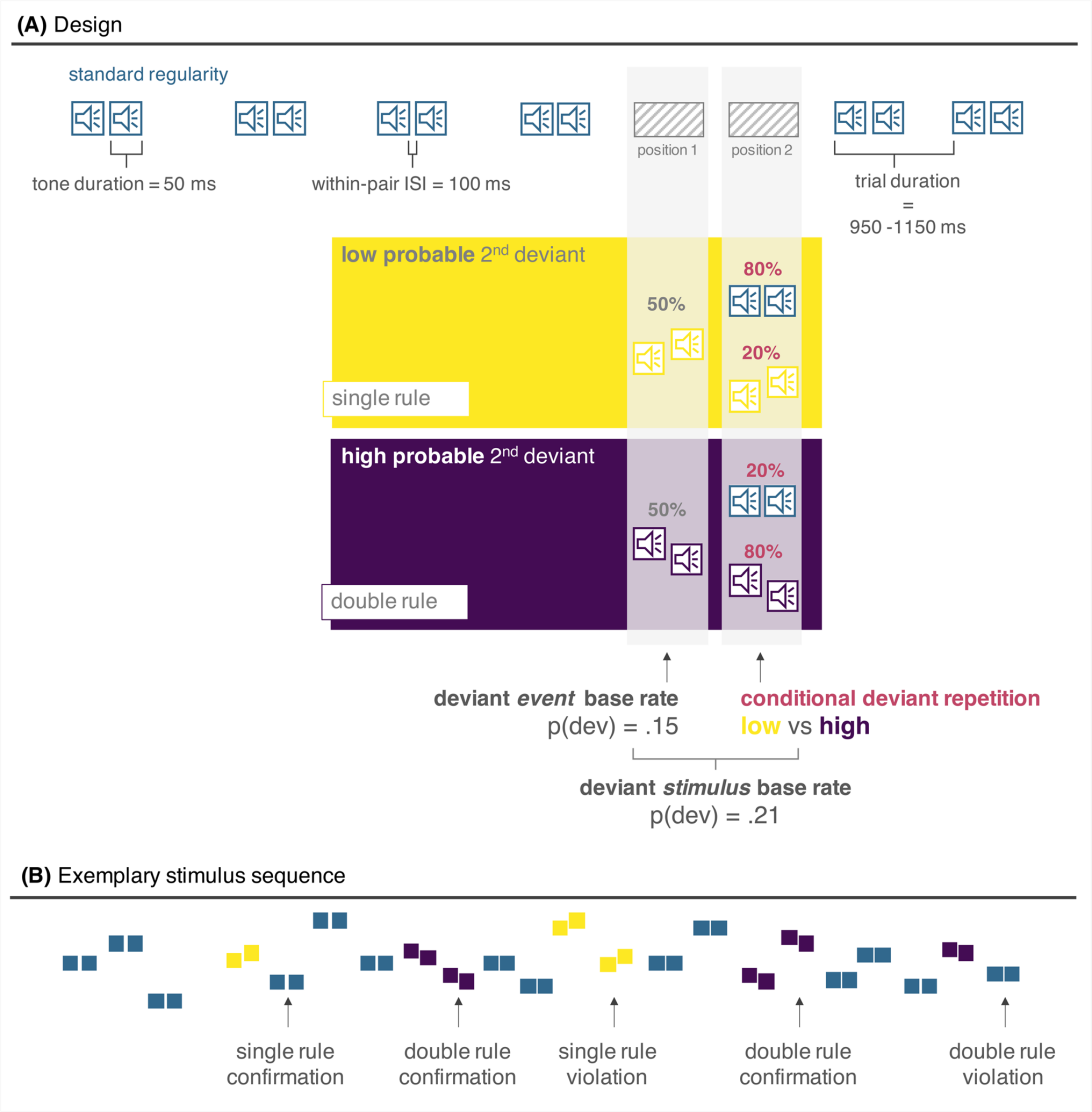
(**A**) Design and (**B**) exemplary auditory stimulation sequence. The sequence was characterized by the occurrence of tone pairs varying in absolute pitch between trials. The standard regularity was defined as a tone pair consisting of two identically pitched tones (constant tone pair). There were two types of deviants: One deviant type had an ascending structure within each pair (pitch of second tone higher than first) and the other deviant type had a descending structure (pitch of second tone lower than first). Counterbalanced across participants, each deviant type was associated with a specific deviant repetition rule: one deviant had a high probability of repetition (purple-coloured stimuli), while the other deviant had a low probability of repetition in the subsequent trial (yellow-coloured stimuli). To extract the regularity of higher order, the direction of within-pair pitch change needs to be extracted, as the absolute pitch of a given tone is not informative about the stimulus’ identity (standard or deviant). Please note that deviant stimulus base rate corresponds to the proportion of trials in which a deviant sound was presented, and deviant event base rate corresponds to the proportion of first deviants within the standard sequence (i.e., the number of repetition rule transitions from a first deviant to the subsequent sound). (Colour figure online)

As can be seen in Fig. 1, sound sequences presented in the main part were constructed based on our recent variation of the classic oddball paradigm (Coy et al., 2022), systematically manipulating conditional deviant repetition probability. The constant pairs (two identical pitches) served as standards, whilst ascending and descending tone pairs occurred as rare deviants following diametrically opposed repetition rules: While one deviant type (e.g., ascending pairs) occurred mostly in two trials in a row (double rule), the other deviant type (e.g., descending tone pairs) was mostly followed by a standard in the subsequent trial (single rule). Only on a subset of trials (20% respectively) each repetition rule was violated: that is, the double rule deviant, instead of repeating (e.g., a second ascending tone pair), was directly followed by a standard. For the single rule, instead of returning to the standard regularity, it was followed by a second deviant of its kind (e.g., a second descending tone pair). Which deviant type follows which repetition rule was counterbalanced across participants. As in our previous use of the paradigm with lower-order regularities (Coy et al., 2022), participants in Experiment 1 were not informed of these oddball repetition rules (naïve).

During the main part, the first five sounds in each block were standards, and at least the first two respective deviant events were rule-conforming. There were always at least two standard trials between deviant events. It was shown that the context in which a given stimulus is first encountered, can shape the processing of that stimulus later, even when the context is changed then (Fitzgerald & Todd, 2020; Mullens et al., 2014; Todd et al., 2011, 2014, 2020). To control for such primacy effects or put more simply, to ensure that the repetition rules can be learned correctly during initial exposure, there were only rule-conforming deviant events in the training block. Each of the 12 experimental blocks consisted of 220 trials, of which 175 were standards (79%) and 45 were deviants (21%; 15 single deviants and 15 deviant repetitions).

#### Data pre-processing and statistical analysis

Data preprocessing was performed offline. Data analysis was run in R using the RStudio environment and a number of packages, mainly from the *tidyverse* (Wickham et al., 2019), such as *dplyr* (Wickham et al., 2021) and *tidyr* (Wickham & Henry, 2020) for data wrangling, *ggplot2* (Wickham, 2016) to generate plots. A repeated-measures analysis of variance (ANOVA) was implemented with *afex* (Singmann et al., 2021) and *emmeans* (Lenth, 2021) was used for follow-up contrasts and estimation of Odds Ratios. Multilevel logistic regression was conducted with *lme4* (Bates et al., 2015) and *lmerTest* (Kuznetsova et al., 2017). Effect sizes were bootstrapped with *bootES* (Kirby & Gerlanc, 2013). Complementary Bayesian analysis was implemented with *BayesFactor* (Morey & Rouder, 2018) and the Bayesian multilevel logistic regression with *brms* (Bürkner, 2017, 2018).

Family-wise error rate of contrasts (i.e., the alpha probability of the multiple tests) was controlled by adjusting *p* values with the Holm method (Holm, 1979). To follow the call for more stringent standards of evidence when claiming new findings, the alpha level was set to 0.005 (Benjamin et al., 2018). In addition to traditional frequentist tests, complementary Bayes factors (*BF*_10_) were calculated as the ratio of evidence given the observed data for the alternative hypothesis, defined as a Cauchy prior distribution centred around 0 with a scaling factor of r = √2/2 (Rouder et al., 2009, 2012), and for the null hypothesis, which corresponded to a standardized effect size δ = 0. The Bayes factor is directly interpretable as an odds ratio (Rouder et al., 2009). That is, for the Bayesian equivalent of a *t* test, it reflects the likelihood of the alternative hypothesis (true effect is different from zero) relative to the null hypothesis (true effect is equal to zero) given the observed data. In the case of ANOVAs and multilevel models, *BF*_10_ reflects the likelihood of the model including the effect of interest relative to the null model given the observed data. This likelihood ratio can be easily inverted by taking the reciprocal (1/BF_10_) to express the likelihood of the null model relative to the model including the effect of interest (*BF*_01_). If not indicated otherwise, reported Bayes factors from ANOVAs reflect the comparison between a given model including an effect of interest and the null model including only a random intercept term for subjects.

Response time (RT) was defined as the time between the onset of the second tone and a key press attributed to that sound. At presentation rates faster than 1 Hz, the attribution of a response to a preceding stimulus can become ambiguous, as a given response can overlap or even occur after a subsequent stimulus event (Bendixen & Andersen, 2013). Therefore, the response time window was specified as follows: A key press was attributed to a sound if it occurred at least 150 ms but no later than 950 ms after the onset of the second tone within a pair (note, that this is 150 ms after the onset of the first tone within a pair, i.e., trial onset, because the response-relevant information only comes with the second tone). Note, that the shorter the SOA the more did the response window span into the subsequent trial. However, response windows never overlapped (i.e., the window never spanned until the onset of the second tone within a pair). All valid key presses that were not attributed to any sound of interest (i.e., first deviant, second deviant, or standard following a first deviant) were not further analyzed (response rate to standards following standards: *Median* = 1.3%; *IQR* = 2.5%). In accordance with signal detection theory (SDT), a button press attributed to a deviant sound (target) was treated as a hit, whereas a button press attributed to a standard sound following a first deviant (nontarget) was treated as a false alarm.

##### Accuracy

As accuracy is measured via a binary outcome variable at the single-trial level, both hit (target trials: 0 = miss, 1 = hit) and false alarm rates (nontarget trials: 0 = correct rejection, 1 = false alarm) were analyzed by means of a multilevel logistic regression, specifically a general linear mixed model with a binomial distribution and a logit link function (Jaeger, 2008). As commonly assumed in repeated-measures designs, observations of the same participant tend to be more similar than observations of two different participants. Multilevel models allow to explicitly represent this dependency in the data via the inclusion of random effects at the participant level. In the context of accuracy, a random intercept term reflects that some participants generally perform better or worse than others. At the level of slopes (e.g., the mean difference between two levels of a categorical predictor) a random effect explicitly models variability in the effect of an experimental manipulation between participants. That is, the manipulation may differentially improve or worsen a participant’s performance. Especially in the context of accuracy this is a concern, because general task performance can limit the effect of an experimental manipulation. For instance, a participant who struggles with the task may have trouble responding to two deviants in a row, thus performance drops from first to second deviant, whereas a participant who is in general very good at the task (i.e., performance is at ceiling) has less room for further improvement and might show smaller differences between factor levels than participants with a generally moderate performance. When specifying a random slope, the residual correlation coefficient between random intercept and random slope (controlled for fixed effects) controls for such ceiling (or floor) effects in the data. Whether the inclusion of a particular fixed effect to the model improved its fit to the data, was tested by means of a likelihood ratio test. We report marginal *R*^*2*^_*m*_ as a measure of the proportion of variability explained by fixed parameters, and conditional *R*^*2*^_*c*_ as the proportion of variability explained by both fixed and random parameters. As parameter estimates of the fixed effects are on the logit-scale, these effects are reported as Odds Ratios (*OR*). We also report relevant contrasts in terms of the actual change in model-implied probabilities, because the changes on the logit-scale are not always intuitively interpretable. We computed complementary Bayesian multilevel models and estimated the Bayes Factors for the corresponding model comparisons from the marginal likelihoods.

The design was characterized by the full combination of the factors Position (first vs second deviant), Deviant Repetition Probability (high vs low), and Actual Repetition (repetition vs nonrepetition). For the analysis of hits, all target (i.e., deviant) trials were included in the analysis. That is, all Position 1 deviants were included irrespective of whether the subsequent stimulus was another deviant or a standard. To test whether deviant repetition rules (double vs single rule) affect target detection of the second relative to the first deviant, we fitted the following models all of which included a random intercept over participants and a fixed intercept as well as a random slope for both predictors over participants: (1) a single predictor model estimating a fixed effect of Position (first and second deviant) only, (2) a single predictor model estimating a fixed effect of Repetition Probability (low vs high) only, (3) an additive model including both predictors, and (4) a model including the Position × Repetition Probability interaction. The additive model was respectively tested against each model that contained only one predictor, and against the model that included the interaction.

False alarms were defined as key presses in response to nontarget sounds (i.e., standards following a first deviant). We investigated the effect of Deviant Repetition Probability (high vs low) on false-alarm rates by testing whether the inclusion of a fixed-effect term of this factor to a multilevel logistic regression model improves model fit compared with a model that includes only a random intercept and a random slope over participants. However, for the frequentist multilevel model the random-effects structure was not supported by the data (singular fit). Therefore, here the inclusion of the fixed effect was tested against a random-intercept only model. The complementary Bayesian multilevel model did include the random intercept though.

##### Response times

Within each participant, the median of all collected response times was used as estimate of central tendency to aggregate the typically right-skewed response time distribution on the subject level. Response times of all first deviants (i.e., irrespective of actual repetition) were collapsed within high and low repetition probability, respectively. Response times of deviant at Position 2 (i.e., actual deviant repetition) were analyzed as a function of repetition probability. By nature of the design, the number of trials is unbalanced in Position 2 deviants: there are more high- compared with low-repetition-probability second deviants. While more trials generally improve parameter estimation precision, when comparing these cells, it is the across-subject variability of the difference score between the cell means and not the within-subject single-trial variability within each cell, that is relevant for statistical analysis. The standards that followed a single deviant (nonrepetition Position 2 trials) were not further analyzed, because (like in in the preceding study; Coy et al., 2022), not all participants produced responses (false alarms) to these stimuli (complete cases for 22 of the 48 participants) and even if they did, there are only a few trials for analysis (*Median*_*high*_ = 6 trials; *Median*_*low*_ = 2 trials). If a first deviant (Position 1) was classified as a miss, the subsequent Position 2 trial was removed from the response time analysis (this pertained to few trials only: *M* = 3.7%; *SD* = 2.3%). A repeated-measures 2 × 2 ANOVA compared the effect of Position (first vs second deviant) and Deviant Repetition Probability (low vs high) on response times for deviant repetitions. The *Position* × *Repetition Probability* interaction effect was deconstructed by a set of contrasts on the estimated marginal means (Searle et al., 1980), probing the effect of Position (first vs second) respectively for both Deviant Repetition Probability rules (high vs low).

##### Control task

In the short survey at the end of the experiment, three participants reported misreading or misunderstanding the instructions for the control task. These participants were removed from the analysis of the control task only. In accordance with signal detection theory, ascending tone pairs were defined as “signal” and descending tone pairs as “noise” in the control task. Sensitivity index *d′* was estimated as the difference in respectively* z*-scored hit minus false-alarm rate. As inverse normal transforms of extreme proportions result in mathematically intractable infinities, data were corrected prior to transformation with the log-linear correction (Hautus & Lee, 2006). A *t* test against zero was used to assess whether discrimination ability was above chance at group level.

## Results

### Accuracy

**Fig 2:**
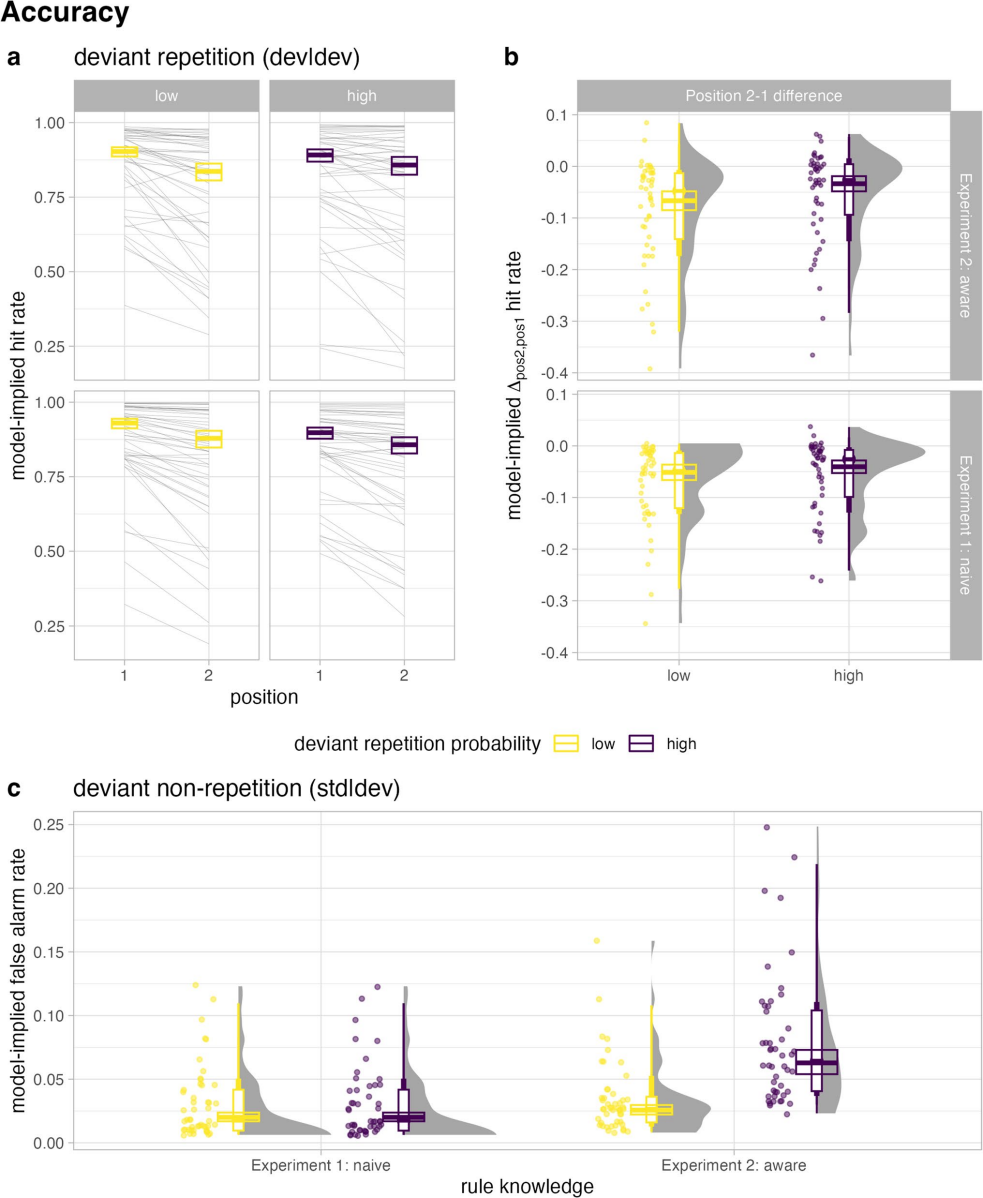
Accuracy. **a–b** Model-implied hit rates (estimated from the Position × Repetition Probability interaction model) in deviant repetition trials as a function of deviant repetition probability and rule knowledge. In the left panel (**a**), hit rates are plotted by stimulus position (first and second deviant). In the right panel (**b**), the respective position 2-1 difference in hit rates is displayed. **c** Model-implied false alarm rates (in Experiment 1 estimated from intercept-only model, in Experiment 2 from the model also including the fixed effect of Repetition Probability) in response to standards following a first deviant (non-repetition) as a function of deviant repetition probability and rule knowledge. The thin grey lines and coloured dots represent the estimated model-implied probability for each subject. Crossbars represent the estimated fixed effect term in each model ±1 *SE*

The respective model-implied probabilities of the accuracy data are provided in Fig. 2 and Table 1.

**Table 1:** Model-implied response rates (hit and false alarm rates) as a function of rule knowledge, deviant repetition probability, and position

	Deviant repetition probability	Position 1		Position 2		∆_21_	
		*M*	*(SE)*	*M*	(*SE*)	*M*	(*SE*)
Exp1: Naïve							
Hit rate	Low	0.930	(0.016)	0.879	(0.028)	−0.051	(0.015)
	High	0.898	(0.019)	0.857	(0.027)	−0.040	(0.012)
False-alarm rate	Low			*0.020*	*(0.003)*		
	High			*0.020*	*(0.003)*		
Exp2: Aware							
Hit rate	Low	0.903	(0.015)	0.836	(0.028)	−0.067	(0.018)
	High	0.891	(0.021)	0.858	(0.030)	−0.034	(0.015)
False-alarm rate	Low			*0.026*	*(0.004)*		
	High			*0.063*	*(0.010)*		

False alarms refer to button presses in response to standards that directly followed a single deviant (nonrepetition). Model-implied probabilities in target trials (hit rates) were estimated from the frequentist model including the fixed effect interaction term of position and repetition probability in each experiment respectively; in non-target trials only the model in Experiment 2 included the fixed effect term for repetition probability. The data in Experiment 1 did not support the inclusion (and thus the estimation) of a fixed slope of repetition probability—therefore, the model-implied false alarm rate in low and high repetition probability is the same and corresponds to the estimated fixed intercept. For the interested reader, we provide a figure of response rate estimates calculated according to signal detection theory on OSF

#### Hits

In the case that two deviants occurred in a row, an additive model (*Deviance* = 19,135, *R*^2^_m_ = 0.016, *R*^2^_c_ = 0.422), including a fixed-effect term for both Position (first vs second deviant) and Repetition Probability (high vs low) fits the data significantly better than a model including Repetition Probability alone (*Deviance* = 19,155, *R*^2^_m_ = 0.007, *R*^2^_c_ = 0.442), χ^2^(1) = 20.13, *p* < .001, *BF*_10_ = 6,664. However, the additive model does not fit the data significantly better than the model including the fixed effect of Position alone (*Deviance* = 19,140, *R*^2^_m_ = 0.008, *R*^2^_c_ = 0.417), χ^2^(1) = 5.53, *p* = .019, *BF*_10_ = 3.9. Including the *Position *× *Repetition Probability* (*Deviance* = 19,128, *R*^2^_m_ = 0.017, *R*^2^_c_ = 0.423) interaction does not significantly improve model fit compared with the additive model, χ^2^(1) = 6.09, *p* = .014, *BF*_10_ = 4.8, but it does significantly better fit the data than the model including the effect of Position alone, χ^2^(2) = 11.62, *p* = .003, *BF*_10_ = 19. Although the odds ratios obtained from the frequentist interaction model slightly differ as a function of repetition probability (low: *OR*_*Pos2,Pos1*_ = 0.546; high: *OR*_*Pos2,Pos1*_ = 0.685) the actual decrease in the model-implied hit rate from first to second deviant is very similar between low (5.1 percentage points, factor of 0.945) and high (4.1 percentage points, factor of 0.954) repetition probability. The reported model-implied hit rates in Table 1, which are also depicted in Fig. 2a–b, were estimated based on the interaction model.

#### False alarms

As can be seen in Fig. 2c (right-hand side) and Table 1, key presses also occurred in response to standards that directly follow a single deviant (deviant nonrepetition)—that is, participants produced false alarms. However, the inclusion of the effect of repetition probability as a fixed term (*Deviance* = 2,252* R*^2^_m_ = 0, *R*^2^_c_ = 0.239) does not significantly improve the model fit over a model including only the random and fixed intercept alone (*Deviance* = 2,252,* R*^2^_m_ = <0.001, *R*^2^_c_ = 0.239), χ^2^(1) = 0.25 , *p* = .617, *BF*_10_ = 0.518. That is, the probability of generating a false alarm in response to a standard does not significantly depend on whether it was preceded by a deviant associated with high compared with low repetition probability (*OR*_high,low_ = 1.1). The model-implied false alarm rates in Table 2 were estimated based on the model that included only the random participants intercept, as the model including the fixed effect of repetition probability was not supported by the data.

**Table 2:** Response times [ms] as a function of rule knowledge, deviant repetition probability and position in deviant repetition

	Deviant repetition probability	Position 1		Position 2		∆_21_	
		*M*	*(SE)*	*M*	(*SE*)	*M*	(*SE*)
Exp1: Naïve							
Deviant repetition	Low	536	(13)	521	(15)	−16	(6)
	High	533	(13)	494	(14)	−39	(7)
Exp2: Aware							
Deviant repetition	Low	524	(10)	511	(14)	−13	(8)
	High	523	(11)	450	(14)	−73	(8)

#### Response times (RT)

**Fig. 3:**
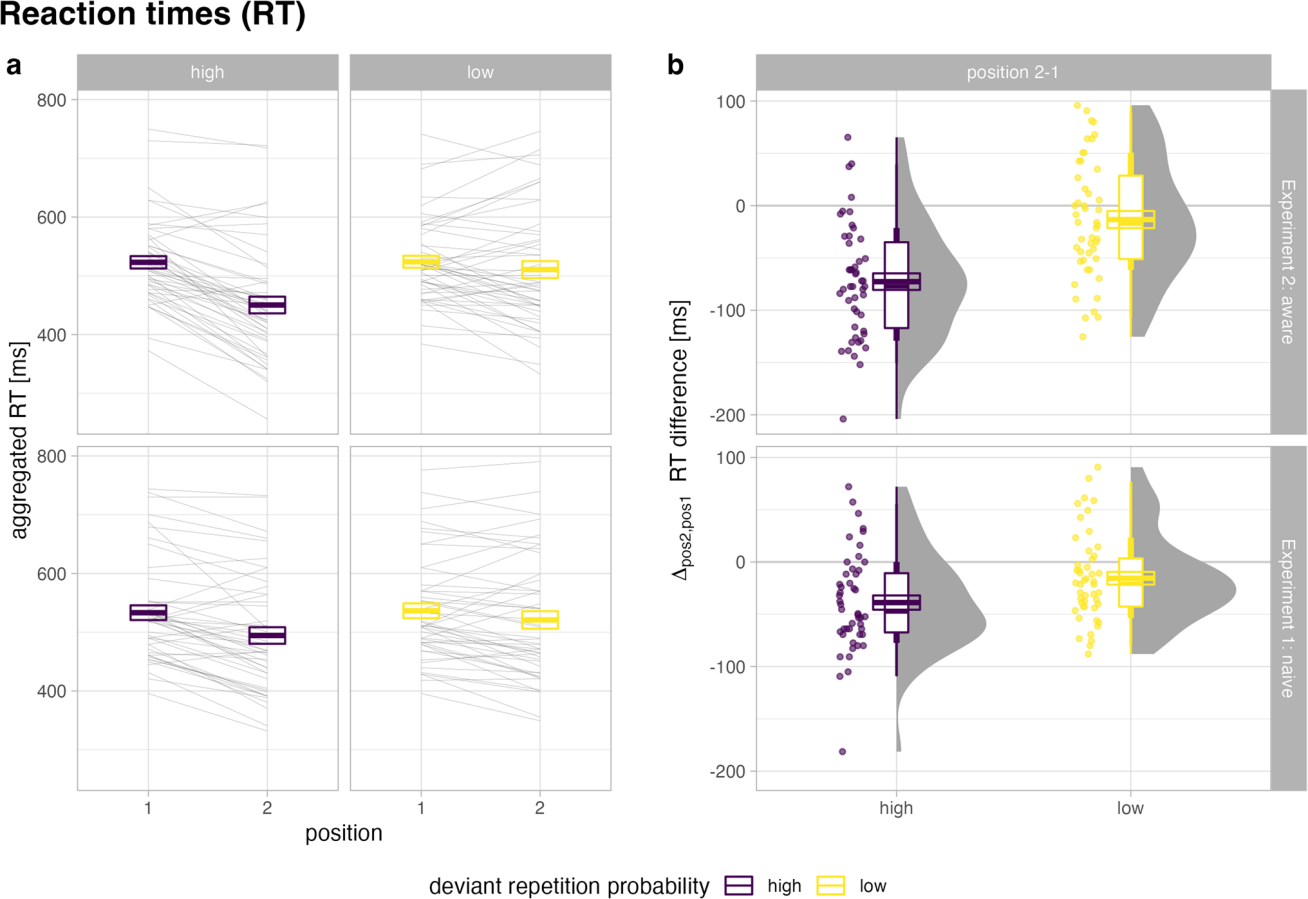
Response times. Aggregated response times (median) in deviant repetition trials as a function of deviant repetition probability and rule knowledge. **a** Response times are plotted by stimulus position (first and second deviant). **b** The respective position 2-1 difference in median response times is displayed. Crossbars represent the mean ±1* SEM*

We show the response time data in Fig. 3 and Table 2. As can be seen in the bottom part of Fig. 3a, median response times on average decrease from first to second deviant (Table 2). While there is both a significant main effect of Position, *F*(1, 47) = 23.09, *p* < .001, *η*_g_^2^ = 0.021, *BF*_10_ = 480 × 10^3^, and of Repetition Probability, *F*(1, 47) = 15.36, *p* < .001, *η*_g_^2^ = 0.006, *BF*_10_ = 10.1, these factors significantly interact (Fig. 3b), Position × Repetition Probability: *F*(1, 47) = 13.78, *p* < .001, *η*_g_^2^ = 0.004, *BF*_10_ = 558 × 10^5^. The model including the interaction effect is preferred over the additive model including both main effects, *BF*_10_ = 4.6. On average, median response times decrease significantly from first to second deviant by 39 ms when repetition probability is high, ∆_21_ *t*(94) = −5.69, *p*_*adj*_ < .001, *d*_z_ = −0.82, *BF*_10_ = 208 × 10^2^, but the descriptive decrease of 16 ms from first to second deviant when repetition probability is low is not significant, Δ_21_ *t*(94) = −2.550, *p*_adj_ = .014, *d*_z_ = −0.37, *BF*_10_ = 2.84.

#### Control task

On average participants were well able to discriminate ascending from descending tone pairs (*d′ M* = 2.08, *SD* = 1.07) in the control task. At group level *d′* was significantly above chance, *t*(44) = 12.966, *p* < .001, *d* = 1.93, *BF*_10_ = 479 × 10^11^.

## Discussion of Experiment 1

As in our preceding study (Coy et al., 2022), the manipulation of repetition rule did not affect the accuracy in the deviant detection task, that is deviant repetitions occurring with high probability were not clearly associated with a higher hit rate than deviant repetitions occurring with low probability. Yet there was a marked effect of position on hit rates, that is on average hit rates declined from first to second deviant. There was a trend in the data that this decline was smaller when the repetition was predictable (high-repetition probability) compared with unpredictable (low-repetition probability), but the actual change at the level of probability was small and thus does not justify a confident inference with regard to successful application of repetition rules at the level of hit rates.

More generally, the decline in hit rates from first to second deviant might be explained by the higher difficulty of discriminating the more complex stimulus set employed in this study. Specifically, to detect a first deviant, participants must discriminate change (ascending or descending) from constancy in relation to the preceding standard. To detect a second deviant, they do not have a constant reference pair in the preceding trial and thus have to rely more on analyzing the individual pair.

Surprisingly, we observed no effect of repetition rules on false alarm rates. To elaborate, in the preceding study false-alarm rates increased by a factor of three when deviant repetition was highly probable compared with when repetition probability was low (Coy et al., 2022), but in the current Experiment 1 we observed no significant difference in false alarm generation as a function of deviant repetition rules. Yet there was a clear effect of deviant repetition probability on response times: There was a significant facilitation from first to second deviant specific to the deviant with a high probability of repetition, but no facilitation when repetition probability was low. Nevertheless, the effect of deviant repetition probability on median response times observed in Experiment 1 was notably smaller compared with the findings in the preceding study (Coy et al., 2022). As the effect of deviant repetition probability seems to mainly manifest at the level of response times (speed), rather than both speed and accuracy, alternative explanations for the observed RT differences need to be considered. Since there are one and a half as many instances of the deviant type that typically repeats compared with the deviant that typically occurs in isolation, a difference in stimulus–response binding (Hommel, 2019; Hommel et al., 2001; Logan, 1988) might have emerged that is driven by global probability (base rate) rather than conditional repetition probability. This may have differentially enforced stimulus–response binding for the more frequent deviant (high-repetition probability) compared with the less frequent deviant (low-repetition probability). Upon correct classification of a deviant stimulus as a target (deviant detection), there could consequently be more facilitation of the response (-selection) component for the deviant with the high compared with the low-repetition probability. This would explain why there is an effect on speed but not on accuracy—because this benefit of stimulus–response binding only emerges when target detection was successful. However, it seems unlikely that observed RT effects are entirely driven by a difference in stimulus-response binding between the two deviant types, because then performance should have been facilitated for the more frequent deviant type irrespective of stimulus position (which was not the case).

Another potential confound, as already discussed in the introduction, is that deviant repetition rules might only be extracted and applied successfully if participants are able to recognize a given deviant type in the first place. If (some) participants were not well able to distinguish the deviant types, this might explain why smaller effects compared with the previous study (Coy et al., 2022) were observed. Yet the control task, in which only ascending and descending tone pairs were presented, shows that participants were able to discriminate ascending from descending tone pairs reasonably well, rendering this explanation unlikely. It should be mentioned, though, that the main experiment and the control task differed in task difficulty because (1) there are three sound types in the former (constant, ascending and descending tone pairs) but only two sound types in the latter (ascending and descending), and (2) SOA was jittered in the main experiment but fixed (and slower) in the control task. But the most important issue is that discriminating the specific direction changes (ascending vs descending) is not the same as distinguishing change from constancy. Although the control task shows that ascending and descending tone pairs can be discriminated, to successfully perform this task, it is sufficient to rely on a same–different comparison in the main experiment. To detect first deviants as such in the main experiment, it is sufficient to identify them as a mismatch to the established standard regularity (are pitches within a pair constant or not constant) but there is no need to identify the actual direction of pitch change. To successfully learn and subsequently apply the repetition rule associated with each deviant type, however, it is crucial to not only recognize a deviant as having two different pitches within a pair, but to recognize the direction of the within-pair pitch change. If participants relied on a strategy that focuses on the constant/nonconstant comparison to detect deviants, the actual pitch direction might not have been salient enough in Experiment 1. On the one hand, this might result in comparatively smaller effects of repetition rule compared with the preceding study (Coy et al., 2022), and on the other hand, it might explain the reduction in hit rates from first to second deviant (see above). That is, a deviant-detection strategy based on constancy of within-pair pitch may yield the representation of the standard regularity less accessible after a first deviant, or the pitch direction change may need to be processed after initial stimulus classification or even upon encountering the subsequent stimulus.

To facilitate that first deviants are processed with regard to the type of deviant (ascending/descending), we decided to directly manipulate awareness of the repetition rules by running a follow-up experiment within a new sample in which participants were made aware of the repetition rules. The advantage of using explicit information of the repetition rules is that it allows to test whether participants are able at all to apply these rules. In Experiment 2, participants received explicit instruction about the repetition rules (which deviant type typically repeats and which deviant typically occurs in isolation). Again, we hypothesized that deviant repetition probability would lead to effects on performance (hit rates, false-alarm rates, response times) as observed in Coy et al. (2022). We extended these hypotheses for Experiment 2 in so far, as that explicit knowledge of deviant repetition rules boosts the effects of deviant repetition probability on performance as described in the introduction.

## Experiment 2

### Methods

#### Participants

Forty-eight people (*M* = 23.2; ranging 18–38 years) participated in Experiment 2, which was also conducted as a web-based experiment. Three additionally tested participants were excluded because they failed to detect at least 50% of either one of the two target types in Position 1 (first deviants) and they responded to more than 10% of the regular standards with a button press (i.e., a false alarm). None of the participants from Experiment 1 took part in Experiment 2.

#### Stimulation

Stimuli, procedure and timing was exactly the same as in Experiment 1. The only difference to Experiment 1 was that participants in Experiment 2 received explicit instructions as to the deviant repetition rules (i.e., “ascending tone pairs mostly repeat and descending tone pairs mostly occur in isolation”, or vice versa).

In Experiment 2 the training block could also be repeated as often as participants wanted before proceeding to the main experiment (38 of the participants performed one, eight performed two; one performed three, and one performed five training blocks).

#### Data pre-processing and statistical analysis

Data preparation and subsequent analysis was identical to Experiment 1. If a first deviant (Position 1) was classified as a miss, the subsequent Position 2 trial was removed from the response time analysis (*M* = 3.0%; *SD* = 2.1%). For hits, to test whether deviant repetition rules (double vs single rule) affected target detection of the second relative to the first deviant, the following models were fitted that all included a random intercept over participants, affixed intercept as well as a random slope for both predictors over participants: (1) a single predictor model estimating a fixed effect of Position (first and second deviant) only, (2) a single predictor model estimating a fixed effect of Repetition Probability (low vs high) only, (3) an additive model including both predictors, and (4) a model including the Position × Repetition Probability interaction. The additive model was respectively tested against each model that contained only one predictor, and against the model including the interaction. The effect of Deviant Repetition Probability (high vs low) on false-alarm rates was investigated by testing whether the inclusion of a fixed effect term of this factor to a multilevel logistic regression model improves model fit compared with a model only including a random and fixed intercept and a random slope over participants.

In addition to the analyses carried out for Experiment 1, further tests were applied on a joint dataset from both experiments to probe whether explicit rule knowledge (Experiment 2: aware) compared with its absence (Experiment 1: naïve) boosts the dissociation of high- and low-repetition probability effects. For false-alarm rates, a restricted model was specified that included a random and fixed effect slope of Repetition Probability and a random participant intercept. The following unrestricted models were tested against the restricted model by means of a Likelihood Ratio test: (1) a fixed effect slope for the factor Rule Knowledge (naïve vs aware) and (2) a fixed effect product term between Repetition Rule and Rule Knowledge. The contrast of interest, reported as Odds Ratio, was between Experiment 1 (naïve) and Experiment 2 (aware) for high and low repetition probability, respectively. For hit rates, the same logic of model comparisons was applied but with the additional terms related to the effect of position. For response times the relevant contrasts were compared between Experiment 1 and 2 by means of an independent *t* test (or nonparametric equivalent). Here, the contrast of interest was the Position 2-1 difference in median response times between Experiment 1 and 2 for high versus low repetition probability, respectively.

In the short survey at the end of the experiment, one participant reported misreading or misunderstanding the instructions for the control task. This participant was removed from the analysis of the control task only.

## Results

### Accuracy

#### Hits

The additive model (*Deviance* = 19,441, *R*^2^_m_ = 0.007, *R*^2^_c_ = 0.384) including both Position (first vs second deviant) and Repetition Probability (high vs low) fits the data significantly better than a model including Repetition Probability alone (*Deviance* = 19,452, *R*^2^_m_ < 0.001, *R*^2^_c_ = 0.391), χ^2^(1) = 11.56, *p* < .001, *BF*_10_ = 252 × 10^67^. However, the additive model does not fit the data significantly better than the model including the fixed effect of Position alone (*Deviance* = 19,441, *R*^2^_m_ = 0.007, *R*^2^_c_ = 0.384), χ^2^(1) = 0.08, *p* = .778, *BF*_10_ = 167 × 10^67^. Including the *Position* × *Repetition Probability* interaction (*Deviance* = 19,430, *R*^2^_m_ = 0.008, *R*^2^_c_ = 0.385) interaction does significantly improve model fit compared with the additive model, χ^2^(1) = 10.46 , *p* = .001, *BF*_10_ = 42. However, the interaction model does not have a significantly better fit than the model including only a fixed slope for the position effect, χ^2^(2) = 10.54, *p* = .0051, *BF*_10_ = 705 × 10^68^. Note, that the equivalent Bayesian multilevel model indicates moderate to strong evidence for the inclusion of the interaction of Repetition Probability and Position. The odds ratios obtained from the frequentist interaction model slightly differ as a function of repetition probability (low: *OR*_*Pos2,Pos1*_ = 0.549; high: *OR*_*Pos2,Pos1*_ = 0.734). The model-implied hit rates from first to second deviant decrease by 6.7 percentage points when repetition probability is low but only by 3.4 percentage points when it is high. Thus, the decline in model-implied hit rates from first to second deviant is smaller when repetition probability is high compared with low. Yet, at the level of model-implied probability, the proportion of detected second relative to detected first deviants differs only slightly as a function of repetition probability (high: factor of 0.963; low: factor of 0.926).

#### False alarms

The inclusion of the effect of repetition probability as a fixed term (*Deviance* = 2,936* R*^2^_m_ = 0, *R*^2^_c_ = 0.190) significantly improves the model fit over a model including the random effect terms alone (*Deviance* = 2,955,* R*^2^_m_ = 0.034, *R*^2^_c_ = 0.203), χ^2^(1) = 19.61, *p* < .001, *BF*_10_ = 5,587. That is, the model-implied probability of generating a false alarm in response to a standard significantly increases by 3.7 percentage points, that is by a factor of 2.4, when the preceding deviant is associated with high compared with low repetition probability (*OR*_*high,low*_ = 2.54).

#### Response times (RT)

As can be seen in the bottom part of Fig. 3a, median responses become faster from first to second deviant (Table 2). While there is both a significant main effect of Position, *F*(1, 47) = 39.05, *p* < .001, *η*_g_^2^ = 0.059, *BF*_10_ = 142 × 10^4^, and of Repetition Probability, *F*(1, 47) = 21.18, *p* < .001, *η*_g_^2^ = 0.031, *BF*_10_ = 310, these factors significantly interact (Fig. 3b), Position × Repetition Probability: *F*(1, 47) = 50.13, *p* < .001, *η*_g_^2^ = 0.029, *BF*_10_ = 194 × 10^11^. The model including the interaction effect is preferred over the additive model including both main effects, *BF*_10_ = 5,693. That is, on average median response times decrease significantly from first to second deviant by 73 ms when repetition probability is high, ∆_21_ *t*(47) = −9.167, *p*_*adj*_ < .001, *d*_z_ = −1.32, *BF*_10_ = 181 × 10^7^, but there is no significant reduction (*M* = −13 ms) of response times from first to second deviant when repetition probability is low, Δ_21_ *t*(47) = −1.626, *p*_adj_ = .111, *d*_z_ = −0.24, *BF*_10_ = 0.532.

#### Control task

On average participants were well able to discriminate ascending from descending tone pairs (*d′ M* = 2.09, *SD* = 0.96). At the group level, *d′* values were significantly above chance, *t*(46) = 14.930, *p* < .001, *d* = 2.18, *BF*_10_ = 169 × 10^14^.

#### The effect of rule knowledge

##### Hit rates

The inclusion of Rule Knowledge (naïve vs aware) in addition to the restricted model (*Deviance* = 38,573* R*^2^_m_ = 0.011, *R*^2^_c_ = 0.399) did not significantly improve model fit to the data (*Deviance* = 38,573* R*^2^_m_ = 0.013, *R*^2^_c_ = 0.400), χ^2^(1) = 0.52, *p* = .472, *BF*_10_ = 0.820. This indicates that there is no evidence for a difference in general deviant detection performance between the two experiments. Model fit was also not improved by additionally including the interaction between Rule Knowledge, Position, and Repetition Probability (*Deviance* = 38,571* R*^2^_m_ = 0.013, *R*^2^_c_ = 0.400), χ^2^(3) = 1.51, *p* = .680, *BF*_10_ = 0.095. Thus, there is no evidence for a differential effect of repetition rules as a function of rule knowledge on hit rates.

##### False-alarm rates

The inclusion of Rule Knowledge (naïve vs aware) as a fixed effect term to the restricted model (*Deviance* = 5,212* R*^2^_m_ = 0.010, *R*^2^_c_ = 0.223) does not significantly improve the fit to the data, additive model (*Deviance* = 5,205* R*^2^_m_ = 0.031, *R*^2^_c_ = 0.239), χ^2^(1) = 6.38 , *p* = .011, *BF*_10_ = 10.4. This indicates that there is no general difference in performance in terms of false alarms between the two experiments. However, including the interaction term between Rule Knowledge and Repetition Probability does significantly improve the model fit, interaction model (*Deviance* = 5,193* R*^2^_m_ = 0.025, *R*^2^_c_ = 0.225), χ^2^(1) = 11.87, *p* < .001, *BF*_10_ = 252. Thus, there is a significant dissociation between high and low-deviant repetition probability only in Experiment 2 but not in Experiment 1: False-alarm rate is relatively comparable when deviant repetition probability is low (*OR*_aware, naïve_ = 1.26), but when repetition probability is high, the false-alarm rate increases when rule knowledge is explicitly available (aware) compared with when it has to be acquired implicitly (naïve), *OR*_aware, naïve_ = 2.97.

##### Response times

There is no significant effect of rule knowledge on the Position 2-1 difference in response times when repetition probability is low, aware versus naïve: *t*(94) = 0.218, *p*_*adj*_ = .828, *d* = -0.045, *BF*_10_ = 0.22. But the decrease in response times from first to second deviant when repetition probability is high is significantly larger when participants are aware compared with when they are naïve to the repetition rules, aware versus naïve:* t*(94) = −3.240, *p*_*adj*_ = .003, *d* = 0.661, *BF*_10_ = 19.

##### Control task

There is no significant difference in the ability to discriminate the two deviant types (ascending vs descending) between Experiment 1 and 2; aware vs naïve: *t*(90) = 0.054, *p* = .957, *d* = 0.011, *BF*_10_ = 0.22.

## Discussion

The human auditory system is believed to operate in a predictive manner, such that regularities inherent in auditory information is represented in some form of internal models. Regularities may be defined by the recurrence of a specific sound or physical feature (lower-order regularities; e.g., all sounds have the same pitch) but also through some common rule that governs stimulus relationships (higher-order regularities; e.g., sounds have varying pitch but are arranged in pairs of the same direction of a within-pair pitch change). Expanding on previous behavioural studies on whether auditory internal models incorporate predictive information from globally unexpected sounds (Berti, 2008; Coy et al., 2022; Parmentier et al., 2011), the current study aimed to test whether these findings largely based on lower-order auditory regularities can also be observed with higher-order auditory regularities. To this end, we modified a recently proposed variation of the classic oddball paradigm (Coy et al., 2022), so that conditional oddball repetition rules were implemented using auditory regularities of higher order. Specifically, two different deviant types (ascending and descending tone pairs of varying absolute pitch; standard regularity was defined by constancy of pitch within a tone pair) were associated with different local probabilities regarding the subsequent sound type (high vs low probability of deviant repetition). As described by Coy et al. (2022), successful extraction of predictive information carried by deviants should reflect both on the level of accuracy and of response times. By directly manipulating knowledge of the repetition rules, we could assess whether explicit knowledge boosts effects compatible with the extraction of these repetition rules.

Generally speaking, the majority of participants was well able to comply with the instructions and managed to respond to targets within the predefined response time window reasonably well, though the task appeared to be more on the challenging side (detection rate of first deviants in the range of 83% to 86%).

### Hypothesis 1: Correct target detection in two successive deviants

As in the preceding study, which employed the conditional oddball repetition paradigm with lower-order pitch repetition regularities (Coy et al., 2022), we observed no clear effect of deviant repetition rule on hit rates with higher-order regularities. Although there was a small benefit in detecting a second deviant when repetition probability is high compared with low in both experiments, this effect was small to negligible at the level of actual change in hit rates. Thus, this should be taken with caution and only tentatively be interpreted as evidence for a benefit of repetition rules at the level of hit rates. While average hit rates were notably lower than in the preceding study (Coy et al., 2022), in which performance was overall near ceiling, there were several participants in both experiments of the current study who showed performance near ceiling as well. Thus, it is quite possible that hit rates generally grant less room than response times, for a potential benefit of the deviant repetition rules to unfold.

Notably though, when a first deviant was followed by a second deviant (deviant repetition), hit rates declined by approximately five to seven percentage points both in participants naïve to (Experiment 1) and aware of (Experiment 2) the deviant repetition rules. This drop in target detection might indicate that to respond to a deviant if the preceding sound also required a response, is generally challenging. However, in the preceding study there was no significant effect of position on hit rates (Coy et al., 2022), indicating that this decline in hit rates from first to second deviant is likely related to the stimulus material, rather than directly attributable to the response repetition.

As also already discussed for Experiment 1, there might have been a confound related to stimulus position. Namely, that for a first deviant it is sufficient to recognize it as different from the standard regularity without the necessity to actually identify its type (ascending vs descending). This information might only be processed after response execution, perhaps interfering with the processing of the subsequent stimulus (Position 2). Not all stimulus properties being directly analyzed in deviant processing was also proposed by Carral et al. (2005). They used similar stimuli (constant standard and nonconstant deviant tone pairs) as employed in the current study, though deviants did not repeat. In their study the second tone of a given deviant pair was either higher (ascending) or lower (descending) than the first tone, and additionally, they varied the magnitude of within-pair pitch change. The authors concluded that initial mismatch processing only reflects that a deviant is a mismatch to the established standard regularity (constant vs nonconstant comparison) and that other attributes (e.g., magnitude of deviation) are analyzed at later stages of auditory processing. This might be exacerbated by the relatively fast stimulation, as on occasion the response to a first deviant occurs only after the onset of the subsequent stimulus. Additionally, the variable length of stimulus-onset asynchrony (SOA) may well have increased task difficulty. In electrophysiological studies it was shown that temporal regularity (fixed compared with jittered SOA) facilitates deviant processing in lower-order regularities (Schwartze et al., 2012; Tavano et al., 2014). This might be also helpful in higher-order regularities. Another explanation for the decline in target detection from first to second deviant could be, that for first deviants there is a clear memory trace of the standard regularity against which the incoming stimulus can be compared. This trace might be less accessible for the second deviant, resulting in poorer detection performance.

### Hypothesis 2: False alarms in response to standards following a single deviant

For standards preceded by a single deviant sound (nonrepetition) we did observe an effect of repetition probability on accuracy. This effect was dependent on whether or not participants were aware of the repetition rules. While there was no evidence of an effect of deviant repetition on false-alarm rates when participants are not informed of the repetition rules (naïve), false-alarm rates increased by more than factor two under high (compared with low) probability of deviant repetition when explicit knowledge was given via instruction (aware). This lends support to the notion that local predictions can in principle be inferred from the conditional repetition information carried by the deviants. It must be stressed, however, that with the higher-order regularities of the current study, deviant repetition rules did not manifest into differential false alarm rates when these rules had to be acquired implicitly. This is an interesting discrepancy with the findings from lower-order regularities (Coy et al., 2022; Parmentier et al., 2011) that behavioural effects (such as facilitation or distraction) emerged without telling participants about the deviant repetition regularities. This suggests that for simple regularities these repetition rules are easier to pick up; thus, explicit prior knowledge is not required in these instances. Whether it would nevertheless increase effect sizes of the repetition rules was not tested for lower-order regularities.

Another contributing factor could be the saliency of the difference between the respective deviant types within a study. In contrast to the preceding study (Coy et al., 2022), pitch of a given sound by itself is insufficient to identify it as either a standard or a deviant. With regard to the higher-order regularity employed in the current study, one can only determine a stimulus as a standard or deviant by extracting the relation between the two tones forming a pair. The direction of a within-pair pitch change might not be as salient in an auditory context in which pitch is constantly changing, as in an auditory context in which altogether only three sounds of different pitch occur. Explicit knowledge of deviant repetition rule might compensate this lack of saliency of higher-order features (here, the direction of pitch change), thus boosting extraction of the deviant repetition rules associated with them.

### Hypothesis 3: Response times in deviant repetition

Lastly, response times (RT) of correctly identified targets were compared between first and second deviants (deviant repetition hit trials) to probe the hypothesized effect of repetition probability. Indeed, response times significantly decreased from first to second deviant when repetition probability was high but not when low—this effect emerged irrespective of whether participants were made aware of or remained naïve to the existence of the repetition rules. This result pattern fits with the preceding study (Coy et al., 2022), demonstrating that response time facilitation is specific to the conditional repetition probability associated with a given deviant. Yet when participants were explicitly informed of the repetition rule, the facilitation from first to second deviant for high deviant repetition probability showed a further increase. That explicit rule knowledge boosts the benefit of predictable information in deviant repetition might be because such top-down information (prior knowledge) provides a predefined framework to organize the sensory experience and also allows for strong priors. Nonetheless, the findings from both Experiments 1 and 2 replicate the specificity of RT facilitation to predictable (high-repetition probability) but not unpredictable (low) deviant repetition.

When taking together the findings from Experiments 1 and 2 of the current study, the observed effects suggest that deviants’ predictive information, at least in response to targets, mainly manifests at the level of detection speed rather than accuracy. Why high-repetition probability translates into an increase of accuracy only when rule knowledge is explicitly available but response time facilitation occurs also in its absence, warrants some further consideration.

One potential confounder could be differential stimulus–response binding (as already elaborated in the discussion of Experiment 1). This is because the deviant type associated with the high repetition probability occurs more often (factor 1.5) than the deviant type associated with the low repetition probability. The extent of potentially differential stimulus–response binding (Hommel, 2019; Hommel et al., 2001; Logan, 1988) contributing to observed performance differences cannot be disentangled with the current data. However, it alone is an insufficient explanation for the observed effects, as one would have expected significant differences of performance also for first deviants as a function of deviant type (which was not the case). Nonetheless, its contribution could be further elucidated in future variations of this conditional oddball repetition paradigm by increasing the number of single deviant trials, such that the global probability of both deviant types is equal—although this would mean that the single rule (deviant nonrepetition) is enforced more often than the double rule (deviant repetition).

Apart from this, a crucial confounding factor is the ability to distinguish the deviant types: To identify a specific direction change (ascending vs descending) is more difficult than (and not the same as) distinguishing change from constancy (tone pair comprised of two identical vs two different pitches). To correctly respond to first deviants, it is sufficient to identify them as a mismatch to the standard regularity, which means that the latter strategy is sufficient. Yet, in order to capitalize on the predictive information related to its repetition probability—that is, whether to anticipate a second target (deviant)—it is necessary to identify the type of deviant (ascending or descending) when encountering the first target (Position 1 deviant). As performance in the control task (in which only ascending and descending tone pairs were presented) shows that ascending and descending tone pairs can be reasonably well discriminated from each other, it seems unlikely that it is a lack of the principle ability to distinguish the deviant types from each other. This was also found in a study using a detection task with similar stimulus material (Schröger et al., 2007). Furthermore, an effect of repetition probability on accuracy did emerge when participants were aware of the repetition rules—which should not occur if deviant types cannot be distinguished.

Therefore, a more likely explanation is in the saliency or perceived relevance of the deviant types. In Experiment 2 (aware) the instruction enforced the distinction of the deviant into an ascending and descending type. However, in Experiment 1 (naïve) instructions only referred to detecting tone pairs that did not consist of two identical (i.e., two different) pitches. This might have inadvertently enforced a strategy in which the discrimination relies upon a constant versus change comparison rather than a processing of the direction of within-pair pitch change when encountering a first deviant. This would also be compatible with the observation that the discrimination performance between ascending and descending tone pairs can be improved with training, even when participants are unable to label the kind of deviation (van Zuijen et al., 2006). One might find alternative ways to make the processing of the deviant types more salient, for instance by changing the within-pair pitch separation, which may improve perceptual discriminability between the deviant types. Yet this would not necessarily address the issue of task strategy focused on a constant/nonconstant comparison. Additionally, if the pitch interval is larger within a tone-pair than between tone-pairs, extracting the within-pair direction of pitch change might actually become more difficult. Therefore, another option would be to make the direction of a within-pair pitch change task-relevant by changing the task from a simple detection task to a two-alternative forced-choice reaction task. That is, by assigning a response key to each deviant type, processing of the pitch direction as opposed to only changing vs constant, might facilitate repetition rule extraction without the need for explicit instruction. Another interesting option would be to ask participants to decide on each trial whether a given stimulus is a standard (constant) or a deviant (nonconstant). This would have the added benefit of requiring a response to all stimuli (not only the deviants) and would thus simplify the distinction whether the expectation of repetitions affects accuracy or bias in responding to deviants. However, it might not solve the issue of task strategy (i.e., deviant type saliency), and the stimulation would probably need to be slower than in the current study to enable participants to respond on every trial. Apart from this, one should bear in mind that the deviant repetition rules are violated in 20% of the cases—that is, on average every fifth instance of a repetition rule is a violation. Especially in a high-change auditory environment (higher-order regularities: absolute pitch of pairs changes on every trial; lower-order regularities: one out of three possible pitches occurs on each trial), increasing the validity of the deviant repetition rules (e.g., to 90%, such that only every tenth instance is a rule violation) might boost the learning of the repetition rules.

Another factor could be that while correct target processing can be boosted by predictive information (response time is shortened), the processing resulting in a missed target might be qualitatively different, such as a momentary lapse of attention, or because of ongoing processing still related to the first deviant. In such instances the auditory system might not be in an optimal state to activate a local prediction or the predictive information is simply available too late to take effect.

Lastly, with regard to Experiment 1 of the current study, it is also difficult to ascertain whether all participants indeed acquired implicit rule knowledge, and if so, at which point in the experiment. It is conceivable that implicit deviant repetition rule extraction takes considerable time with a complex higher-order regularity (which in addition is only 80% valid in the main part of the experiment). Assuming that participants acquired rule knowledge after they were well into Experiment 1, would provide a simple explanation for the reduced effect sizes relative to Experiment 2. As the experiments were conducted online, it was—even with the survey at the end—more challenging to get an impression about participants’ possible implicit knowledge about the regularities than it would be in a lab study with direct interaction. The design of future studies could be optimized towards examining learning trajectories for the implicit acquisition of higher-order repetition regularities.

## Towards a more integrative perspective on oddballs

Previous work has meticulously dissected the role of standard sounds in establishing generative or internal models, providing insights into the degrees of complexity that can still be extracted and maintained by our auditory system (for review, see Paavilainen, 2013; Winkler, 2007). There is accumulating evidence supporting the idea of the (human) brain as a Bayesian predictive machine (Clark, 2013; Friston, 2010; Kanai et al., 2015; Knill & Pouget, 2004; Wacongne et al., 2011; Winkler & Czigler, 2012). One common assumption is that the brain, including the auditory system, strives to minimize prediction error by constantly and recursively comparing internal models with sensory input, feeding inconsistencies backwards as prediction error to update higher representations (Garrido et al., 2009; Kanai et al., 2015; Winkler & Czigler, 2012). This is to generate and optimize hypotheses about upcoming information (i.e., the imminent future). Although deviants are typically related in their function to the maintenance of such models, to our knowledge they are usually not assumed to have a direct representation in the model themselves. For instance, the Auditory Event Representation System (AERS) theory (Schröger et al., 2014; Winkler & Schröger, 2015) considers the function of deviant detection to flag sounds carrying information not (yet) covered by the currently active model, triggering additional processes such as adjustment of model confidence, selection, reactivation, update, or even generation of a new model. Which of these options are selected under which conditions and what exactly happens to the underlying model appears to be explained best within a prediction-based account (for review: Winkler, 2007; Winkler & Czigler, 2012). While there are several electrophysiological studies probing whether the deviant can inform subsequent stimulus processing (Deacon et al., 2000; Müller et al., 2005a, 2005b; Rosburg et al., 2018; Sams et al., 1984; Sussman & Winkler, 2001; Tavano et al., 2014; Todd et al., 2010; Todd & Mullens, 2011; Todd & Robinson, 2010), reported effects are somewhat inconsistent. Thus, the role of the deviant within predictive models is not yet well understood. The systematic manipulation of conditional repetition probability information carried by deviant stimuli employed here, provides a new promising paradigm to study deviant processing in this respect. Like the classic oddball paradigm, our variation cannot only be used to study processing in an active listening task as in the current study (i.e., deviant detection), but it can also be combined with another primary task related to the auditory stimulation (e.g., tone duration discrimination task) to study processing during indirect listening (distraction paradigm), or the paradigm can be used in a passive listening situation in which attention is directed to some unrelated task (e.g., watching a video, performing a visual *n*-back task). The degree to which repetition rules inform the underlying model, or the content or strength of predictions inferred from it, likely depends on the current goals—are sounds to be ignored, or do repetition rules directly or indirectly serve the current task? Studying how behavioural and electrophysiological processing related to repetition rules is affected in different attentional settings can help understand how the auditory system incorporates and uses predictive information.

The behavioural data gathered here provide further evidence that local predictions based on deviants can be extracted when directly task-relevant and translate into measurable behavioural effects, even when those predictions are based on higher-order regularities. Yet at this point it is not entirely clear whether top-down explicit rule knowledge is required, or whether task-related strategies (focus on constancy vs change rather than deviant type) determine extraction and application of deviant-related rules. Nonetheless, the current findings provide further evidence that deviant-related effects on behavioural goals are not an inherent attribute of a stimulus’ novelty, deviancy or frequency but reflect match or mismatch to predictions inferred from local contingencies (Parmentier et al., 2011). Whether a deviant’s local repetition probability also informs the internal model when auditory stimuli are outside of direct attention, as in passive listening, remains to be seen. To conclude, the deviant is undoubtedly a powerful tool to make predictions fail and elicit distraction. Yet, in many settings, deviant stimuli do carry information that allows to predict impending auditory events. Broadening the perspective of the deviant as a potential part of the internal models, provides an additional angle to improve our understanding of the predictive properties of (auditory) perception.

## Data Availability

Materials for experimental stimulation, data that support the findings of this study and the scripts used for statistical analysis are openly available in the OSF Repository (10.17605/OSF.IO/42CF3).

## Declaration

### Research transparency

We report how we determined our sample size, all data exclusions (if any), all experimental manipulations, and all measures in the study.
